# Dissociating selectivity adjustments from temporal learning–introducing the context-dependent proportion congruency effect

**DOI:** 10.1371/journal.pone.0276611

**Published:** 2022-12-13

**Authors:** Michael Sprengel, Miriam Tomat, Mike Wendt, Sven Knoth, Thomas Jacobsen

**Affiliations:** 1 Experimental Psychology Unit, Helmut-Schmidt-University/University of the Federal Armed Forces Hamburg, Hamburg, Germany; 2 Faculty of Human Sciences, ICAN Institute for Cognitive and Affective Neuroscience, Medical School Hamburg, Hamburg, Germany; 3 Computational Statistics, Helmut-Schmidt-University/University of the Federal Armed Forces Hamburg, Hamburg, Germany; Southwest University, CHINA

## Abstract

The list-level proportion congruency effect (PCE) and the context-specific PC (CSPC) effect are typical findings in experimental conflict protocols, which competing explanations attribute to different mechanisms. Of these mechanisms, stimulus-unspecific conflict-induced selectivity adjustments have attracted the most interest, from various disciplines. Recent methodological advances have yielded an experimental procedure for entirely ruling out all stimulus-specific alternatives. However, there is a stimulus-unspecific alternative–temporal learning–which cannot even be ruled out as the sole cause of either effect with any established experimental procedure. That is because it is very difficult to create a scenario in which selectivity adjustments and temporal learning make different predictions–with traditional approaches, it is arguably impossible. Here, we take a step towards solving this problem, and experimentally dissociating the two mechanisms. First, we present our novel approach which is a combination of abstract experimental conditions and theoretical assumptions. As we illustrate with two computational models, given this particular combination, the two mechanisms predict opposite modulations of an as yet unexplored hybrid form of the list-level PCE and the CSPC effect, which we term context-dependent PCE (CDPCE). With experimental designs that implement the abstract conditions properly, it is therefore possible to rule out temporal learning as the sole cause of stimulus-unspecific adaptations to PC, and to unequivocally attribute the latter, at least partially, to selectivity adjustments. Secondly, we evaluate methodological and theoretical aspects of the presented approach. Finally, we report two experiments, that illustrate both the promise of and a potential challenge to this approach.

## Introduction

Experimental conflict protocols, like the Stroop task [1,2] or the Eriksen flanker task [3,4] are commonly used to investigate cognitive control. In such protocols, responses to the target stimulus are typically slower and more error-prone in incongruent trials, where distractor stimuli indicate an incorrect response, than in congruent trials, where distractors indicate the correct response. This congruency effect decreases as the list-wide proportion of congruent trials (proportion congruency; PC) decreases [5–7]. Stimulus-unspecific conflict-induced selectivity adjustments are the most prominent explanation for this list-level Proportion Congruency Effect (PCE) [8,9]: According to the seminal Conflict Monitoring Theory (CMT) [8], the processing system tracks the average level of response conflict, which indicates how much the distractors generally interfere with response selection. The more they interfere (i.e., the lower the PC), the more target processing is prioritized over distractor processing (i.e., the more selectivity increases), in general.

While this mechanism has attracted great interest from various disciplines, in many cases, it is not the only possible explanation for the effect [10,11]. Due to the limited number of stimuli, in many investigations, the list-wide PC correlates positively with the relative frequency with which the individual stimuli occur in congruent trials. Therefore, adaptations to these stimulus-specific PCs would explain the list-level PCE as well [12,13]. Relatively recent methodological advances have yielded an experimental procedure for entirely ruling out such alternatives [11]: So-called diagnostic trials are interspersed between PC-manipulated so-called inducer trials. The PCs of the diagnostic stimuli (i.e., the stimuli presented in diagnostic trials) are the same under conditions of both high and low list-wide PC. Therefore, a PCE in diagnostic trials cannot be the effect of adaptations to these stimulus-specific PCs.

However, there is a stimulus-unspecific mechanism that cannot be ruled out in this way: According to the temporal learning account [12], the cognitive system learns to expect the time of response initiation, over the course of the experiment. In each trial, it strategically transiently reduces the response threshold at the currently expected time. Therefore, the more similar the expected time is to the actual time of response initiation, the better performance is. When the list-wide PC is low, the expected time is more similar to the (average) response initiation time in incongruent trials than when the list-wide PC is high. Therefore, incongruent trials have a smaller processing disadvantage in the former case (i.e., a list-level PCE) [12].

This means that, even if a PCE is found in diagnostic trials, it cannot be unequivocally attributed to stimulus-unspecific selectivity adjustments. In fact, if temporal learning is not controlled statistically [12,14], it cannot be ascertained whether selectivity adjustments have even contributed to the effect. But statistical control is difficult because it is unclear how exactly the temporal expectation should be quantified [12]. It would be much more convenient if temporal learning could be ruled out experimentally, ideally entirely. However, no established experimental procedure even permits the exclusion of temporal learning as the sole cause of a list-level PCE.

In 2017, Schmidt introduced an experimental procedure with a closely related purpose [15]. On a substantial portion of both congruent and incongruent (inducer) trials, he asked participants to withhold their response until a visual wait cue disappeared. He found that when the waiting period eliminated response time differences between congruent and incongruent (inducer) trials and thus the overall response time (RT) difference between the high- and the low-PC condition, the PCE (in diagnostic trials) disappeared. The author interpreted this pattern in favor of the temporal learning account. He acknowledged, however, that it cannot be ruled out that withholding the response interfered with selectivity adjustments through cognitive control [15]. In that case, the absence of the PCE in the absence of an RT difference between the PC conditions would not indicate that temporal learning is responsible for the presence of a PCE in the presence of such an RT difference. There is at least one way in which postponing the response may have interfered with selectivity adjustments which Schmidt (2017) [15] did not mention: Frequently having abundant time to decide/accumulate evidence may make the effortful maintenance of global selectivity settings seem like a bad investment of resources to the cognitive system, especially in the low-PC condition (in which the maintenance of such settings should be especially effortful).

This illustrates how difficult it is to even create a scenario in which selectivity adjustments predict an effect while temporal predicts a null effect. It is even more difficult to create the arguably more desirable scenario in which the mechanisms predict opposite outcomes that are both different from zero. With established experimental protocols designed to quantify the traditional list-level PCE, it appears impossible to create such a scenario.

Another case of stimulus-unspecific adaptations to PC [9] in which established protocols cannot distinguish between selectivity adjustments and temporal learning is the context-specific PC (CSPC) effect [13,16–34]. It refers to the observation that the congruency effect is smaller in a context (i.e., a subset of trials defined by a contextual feature) with lower PC than in a randomly intermixed, and therefore temporally overlapping, other context with higher PC [5,9–11,35]. As the list-level PCE, the CSPC effect has been shown to transfer to PC-unmanipulated diagnostic stimuli [22,23], and can therefore be regarded as stimulus-unspecific. As in the case of the list-level PCE, both conflict-induced selectivity adjustments and temporal learning could be responsible for a CSPC effect in diagnostic trials [10,12,31]. As in the case of the list-level PCE, with established protocols designed to quantify the traditional CSPC effect, it appears impossible to create a scenario in which selectivity adjustments and temporal learning predict opposite outcomes.

In this article, we introduce an as yet unexplored hybrid form of the list-level PCE and the CSPC effect which does allow for the creation of such a scenario. We term this “new” stimulus-unspecific effect “context-dependent PCE (CDPCE)”. As we show, given a combination of abstract experimental conditions and theoretical assumptions, selectivity adjustments and temporal learning predict opposite modulations of this effect. Thus, extending Schmidt’s (2017) [15] efforts, we here take a further step towards experimentally dissociating selectivity adjustments from temporal learning.

It is important to note that, despite our focus on experimental methodology, this is a theoretical article. In the first section, we describe our novel approach for dissociating selectivity adjustments from temporal learning, which is purely theoretical. This approach is the above-mentioned combination of conditions and assumptions given which the two mechanisms predict opposite modulations of the CDPCE. For illustration purposes, we show that computational models of conflict-induced selectivity adjustments and temporal learning produce the expected data patterns in simulations that implement this combination of conditions and assumptions. In the second section, we evaluate methodological and theoretical aspects of the presented approach. Finally, we report two experiments that, though not designed for this purpose, illustrate both the promise of and a potential challenge to this approach.

## Conditions and assumptions

In this section, we describe our approach for dissociating selectivity adjustments from temporal learning. First, we introduce conditions under which the CDPCE and modulations of it can be observed. Then, we introduce two assumptions under which such modulations would occur. Finally, we explain why selectivity adjustments and temporal learning predict opposite modulations of the CDPCE, given the introduced combination of conditions and assumptions. To illustrate how the mechanisms produce these opposite modulations, we present simulations of conflict monitoring and temporal learning that implement the combination of conditions and assumptions.

### Conditions–The Context-Dependent Proportion Congruency Effect (CDPCE)

**Fig 1:**
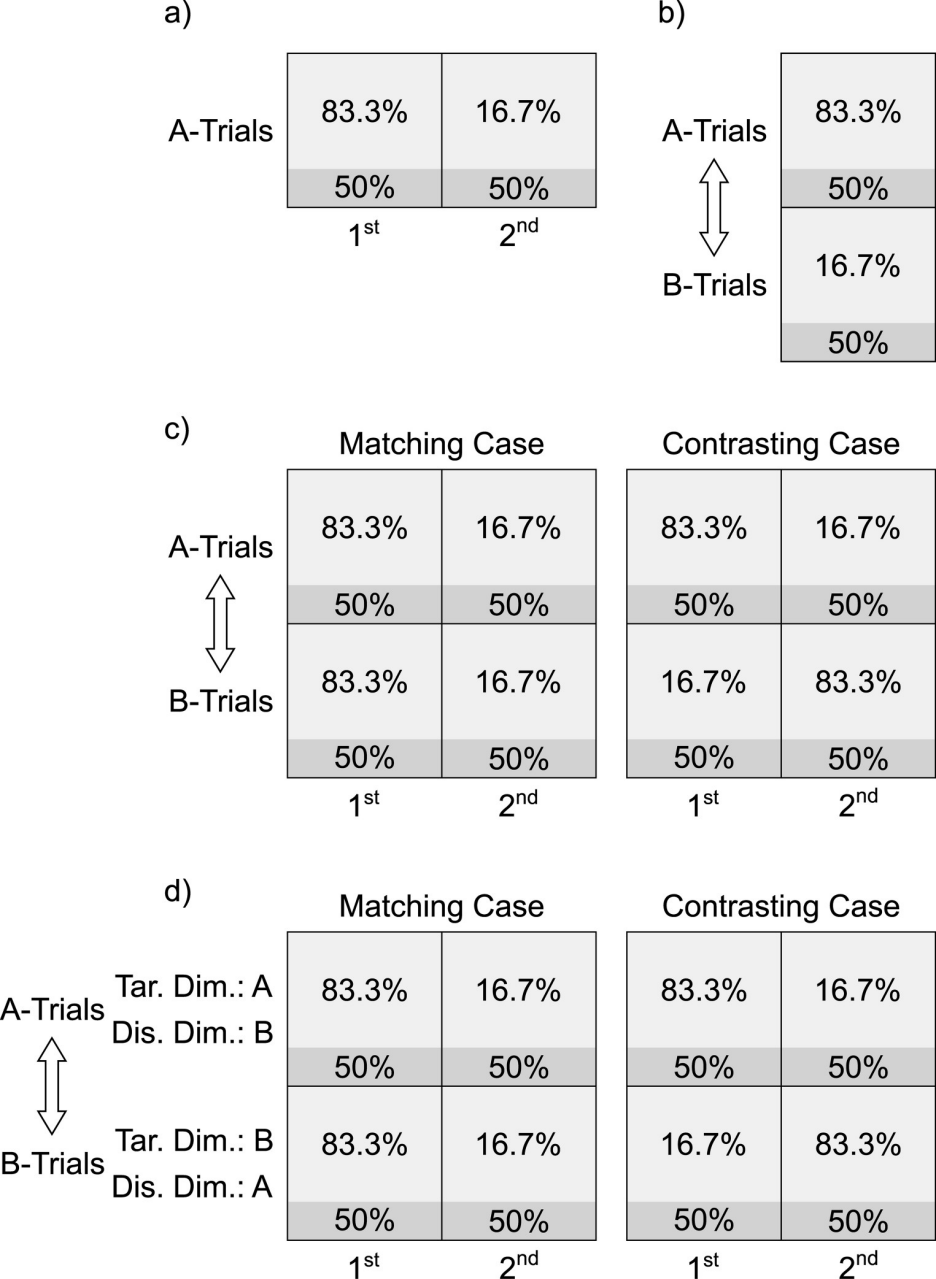
Protocol overview. The A-trials and B-trials are two different contexts. The double-headed arrow indicates frequent switches between these contexts. The percentages are context- and trial-type-specific PCs: Areas in lighter gray represent inducer trials. Areas in darker gray represent diagnostic trials. The size of each area represents the proportion of trials of the respective type, in the respective square field (i.e., 75% are inducer trials and 25% diagnostic trials). “1^st^” and “2^nd^” stand for the first and second half of the respective trial sequence. “Tar.”, “Dis.” and “Dim.” stand for “Target”, “Distractor” and “Dimension”.

Fig 1A illustrates a typical type of protocol designed to demonstrate transfer of the list-level PCE from inducer to diagnostic trials. The transferred PCE in the diagnostic trials (=: Cong_A1 –Cong_A2) is calculated within the only existing context (A-trials) as the difference between the congruency effect in the diagnostic trials in the first half of the trial sequence (=: Cong_A1), the high-PC-condition, and the congruency effect in the diagnostic trials in the second half of the trial sequence (=: Cong_A2), the low-PC-condition.

Fig 1B illustrates a typical type of protocol designed to demonstrate transfer of the CSPC effect from inducer to diagnostic trials. The transferred CSPC effect in the diagnostic trials (=: Cong_A–Cong_B) is the difference between the congruency effect in the diagnostic trials among the A-trials (=: Cong_A), the high-PC-condition, and the congruency effect in the diagnostic trials among the B-trials (=: Cong_B), the low-PC-condition. The double-headed arrow indicates that participants have to switch back and forth randomly between A- and B-trials.

Fig 1C illustrates two cases in which the CDPCE can be computed. In both the Matching case and the Contrasting case, the CDPCE is the average PCE calculated within each of the contexts, that is, the average of the PCE in the diagnostic trials among the A-trials and the PCE in the diagnostic trials among the B-trials. In the Contrasting case, this would be ((Cong_A1 –Cong_A2) + (Cong_B2 –Cong_B1))/2, which is actually the same as the average CSPC effect, in that case, because ((Cong_A1 –Cong_A2) + (Cong_B2 –Cong_B1))/2 = ((Cong_A1 –Cong_B1) + (Cong_B2 –Cong_A2))/2. In the Matching case, however, the traditional CSPC effect cannot be calculated, as the contexts have the same PC in each half of the trial sequence. By contrast, the CDPCE can be calculated, namely as ((Cong_A1 –Cong_A2) + (Cong_B1 –Cong_B2))/2. Focusing on the PCE within each context, in both cases, makes it possible to directly compare the Matching case with the Contrasting case in one analytic design. Thus, it is possible to analyze the PCE within each context as a function of whether or not the PC in that context matches or contrasts with the PC in the respective other context, in each half of the trial sequence. Statistically, a difference in the CDPCE between the Matching and the Contrasting case would manifest in a three-way interaction between congruency, PC and case, across both contexts. Such a difference can reveal subtle reciprocal influences between the contexts that neither the list-level PCE, across both contexts, nor the CSPC effect can reveal. Such reciprocal influences are what our approach is designed to exploit.

Fig 1D illustrates the type of protocol that incorporates all of the abstract experimental conditions that this approach requires:

- A Matching and a Contrasting case
- The target and distractor dimensions are reversed between the two contexts involved in the Matching and Contrasting case. This means that, in the A-trials, dimension A contains the target while dimension B contains the distractor. By contrast, in the B-trials, B contains the target while A contains the distractor.

Due to the latter dimension reversal between the contexts, selectivity adjustments predict a larger CDPCE in the Contrasting case than in the Matching case while temporal learning predicts the opposite size ratio–under the assumption of a reciprocal influence between the contexts, as described in the next section.

### Assumptions

The necessary assumptions for this contrast in predictions are:

- The cognitive system tracks the PC for each context and tries to implement the appropriate adaptations to these context-specific PCs.

The CSPC effect shows that the system can track the PCs of different contexts, and switch between adaptations to these PCs [24–26,29,36].

- What adaptations the cognitive system tries to implement in each context not only largely determines what adaptations are actually implemented in that context but, less strongly, also what adaptations are implemented in the respective other context.

One possible cause of such an interdependence is that the system does not fully reverse context-specific settings when switching to the respective other context. Indeed, the results of a number of relatively recent studies can be interpreted in terms of such carry-over effects. Fernandez-Duque and Knight [37] failed to observe a PCE transfer from a PC-manipulated (i.e., involving different PCs) Stroop task involving color targets to a PC-unmanipulated number Stroop task involving number targets. In contrast, Funes et al. [38], (see also [39], but see [40], for a different result) did observe a PCE transfer from a PC-manipulated Spatial Stroop task involving symbol targets to a PC-unmanipulated Simon Task involving the same symbol targets. Similarly, Wühr et al. [41] only observed a PCE transfer from a PC-manipulated horizontal/vertical Simon task to a PC-unmanipulated vertical/horizontal Simon task when both tasks involved the same target dimension. Furthermore, they observed a PCE transfer from a PC-manipulated Simon/Stroop task to a PC-unmanipulated Stroop/Simon task, which involved the same target dimension but different stimulus-response mappings. All these findings are compatible with carry-over-effects of selectivity adjustments: Adjustments of target processing, in the PC-manipulated context, could be partially carried over to the PC-unmanipulated context. Here, they only become effective (in a similar way), however, if the target dimension is the same as in the PC-manipulated context [41].

Note that, while the reported results are most readily interpretable in terms of interdependent selectivity adjustments, the assumption of interdependence concerns all kinds of adaptations. It is, for example, just as easily conceivable that temporal expectations are partially carried over to another context.

In accordance with the above introduced focus on the PCE within each context, the assumptions concern the operation of the underlying mechanism (i.e., conflict-induced selectivity adjustments or temporal learning) within each context. We start from the assumption that the processing dynamic of the respective mechanism–essentially constituted by the tracking of the respective covariate of PC (i.e., conflict or time of response initiation) and the implementation of adaptations to the tracked values (i.e., selectivity adjustments or shifts in temporal expectation)–could, in principle, be instantiated independently in each context. The second assumption restricts this independence with respect to the implemented adaptations: What would be the appropriate adaptation to the (covariate of) PC in one context influences what adaptation is implemented in the respective other context.

Suppose, for example, that the (according to some criterion) appropriate adaptation to the conflict level tracked for the A-trials is selectivity adjustment X, and the appropriate adaptation to the conflict level tracked for the B-trials is selectivity adjustment Y. Numerically, a reciprocal influence with respect to the actually implemented selectivity adjustment in each context could be represented as a weighted average: For example, in the A-trials, where adjustment X would be appropriate, the actually implemented adjustment could be .75*X + .25*Y. Correspondingly, in the B-trials, where adjustment Y would be appropriate, the actually implemented adjustment could be .75*Y + .25*X. Please note that we do not make any assumptions about how the (weaker) influence by the adaptation appropriate for the respective other context may arise, just that there is such an influence. (For a conceivable scenario, see the discussion of carry-over effects above.)

What is critical about our approach is that the reversal of the target and distractor dimensions between the contexts causes the described weighted average to behave very differently in the case of selectivity adjustments than in the case of shifts in temporal expectations, as described below.

### Opposing predictions

#### Selectivity adjustments

Selectivity adjustments change the degree to which target processing is prioritized over distractor processing. They can involve relative enhancement or inhibition of the processing of target- or distractor-related information at any processing stage(s) from early stimulus perception to response execution. Numerically, they can be expressed as processing weight adjustments. For example, the tuple (1, -1) could represent an increase, represented by 1, of the processing weight of dimension A and a decrease, represented by -1, of the processing weight of dimension B. Correspondingly, the tuple (-1, 1) would represent a decrease of the processing weight of A and an increase of the processing weight of B. Which of these tuples represents a selectivity increase and which a selectivity decrease depends on which dimension contains the target and which the distractor. In the A-trials, dimension A contains the target while B contains the distractor. Therefore, the overall processing weight of A is higher than that of B, and (1, -1) represents a selectivity increase while (-1. 1) represents a selectivity decrease. By contrast, in the B-trials, B contains the target while A contains the distractor. Therefore, the overall processing weight of B is higher, and (-1, 1) represents a selectivity increase while (1, -1) represents a selectivity decrease. Crucially, this means that each adjustment (i.e., each tuple) represents a selectivity increase in one context but a selectivity decrease in the respective other context.

This is crucial because it means that matching context-specific selectivity adjustments are incompatible with each other but contrasting ones are compatible with each other. For example, suppose that the appropriate adjustment to low PC (relative to the selectivity setting [target weight, distractor weight] appropriate for medium PC) in the A-trials is (1, -1) (i.e., a selectivity increase, see above) and the appropriate adjustments to low PC (relative to the selectivity setting [distractor weight, target weight] appropriate for medium PC) in the B-trials is (-1, 1) (i.e., a selectivity increase, see above). Suppose that, correspondingly, the appropriate adjustment to high PC is (-1, 1) in the A-trials and (1, -1) in the B-trials. Applying the above introduced weighted average, when the PC is low in both contexts, the adjustment implemented in the A-trials is .75*(1, -1) + .25*(-1, 1) = (.5, -.5). When the PC is high in both contexts, the adjustment implemented in the A-trials is .75*(-1, 1) + .25*(1, -1) = (-.5, .5). Thus, in the Matching case, the implemented adjustment only has half the size it is “supposed” to have, in both PC conditions. As a consequence, the difference in processing selectivity between the low-PC and the high-PC condition only has half the size it is supposed to have, namely ((target weight, distractor weight) + (.5, -.5))—((target weight, distractor weight) + (-.5, .5)) = (1, -1) instead of ((target weight, distractor weight) + (1, -1))—((target weight, distractor weight) + (-1, 1)) = (2, -2).

By contrast, in the Contrasting case, the adjustments keep their full size. When the PC is low in the A-trials and high in the B-trials, the adjustment implemented in the A-trials is .75*(1, -1) + .25*(1, -1) = (1, -1). Correspondingly, when the PC is high in the A-trials and low in the B-trials, the adjustment implemented in the A-trials is .75*(-1, 1) + .25*(-1, 1) = (-1, 1). As a consequence, the difference in processing selectivity between the low-PC and the high-PC condition keeps its full size of ((target weight, distractor weight) + (1, -1))—((target weight, distractor weight) + (-1, 1)) = (2, -2).

As we have seen, in the Matching case, the difference in processing selectivity between the PC conditions has half the size of that in the Contrasting case. Since the difference in selectivity is the only difference (in the processing mode in the diagnostic trials) between the PC conditions, its size determines the size of the PCE, within each context. Assuming a more or less linear relationship between the difference in the processing mode and the PCE, the CDPCE in the Contrasting case should be roughly twice as large as that in the Matching case.

### Temporal learning

The temporal learning account, on the other hand, predicts a larger CDPCE in the Matching case than in the Contrasting case. That is because shifts in the temporal expectation are independent of which dimension contains the target and which the distractor and therefore have the same effect in both contexts.

For example, suppose the appropriate shift in the temporal expectation when the PC is low (relative to the temporal expectation [reference expectation] when the PC is medium) in the A-trials is 1 (i.e., the response is expected later) and the appropriate shift when the PC is low in the B-trials is also 1 (because the response is also expected later). Suppose that, correspondingly, the appropriate shift when the PC is high is -1 (i.e., the response is expected earlier), in both contexts. Applying the familiar weighted average, when the PC is low in both contexts, the adjustment implemented in the A-trials is .75*1 + .25*1 = 1. When the PC is high in both contexts, the implemented adjustment in the A-trials is .75*-1 + .25*-1 = -1. Thus, in the Matching case, the implemented adjustment has the full size, in both PC conditions. As a consequence, the difference in the temporal expectation between the low-PC and the high-PC condition keeps the full size, namely (reference expectation + 1)—(reference expectation—1) = 2.

By contrast, in the Contrasting case, the shifts have opposite directions and therefore reduce each other to half the size they should have. When the PC is low in the A-trials and high in the B-trials, the adjustment implemented in the A-trials is .75*1 + .25*-1 = .5 Correspondingly, when the PC is high in the A-trials and low in the B-trials, the adjustment implemented in the A-trials is .75*-1 + .25*1 = -.5. As a consequence, the difference in the temporal expectation between the low-PC and the high-PC condition has only half the size it is supposed to have, namely (reference expectation + .5)—(reference expectation—.5) = 1, instead of (reference expectation + 1)—(reference expectation—1) = 2.

As we have seen, in the Contrasting case, the difference in the temporal expectation between the PC conditions has half the size of that in the Matching case. Since the difference in the temporal expectation is the only difference (in the processing mode in the diagnostic trials) between the PC conditions its size determines the size of the PCE, within each context. Assuming a more or less linear relationship between the difference in the processing mode and the PCE, the CDPCE in the Matching case should be roughly twice as large as that in the Contrasting case.

Thus, as we have seen, given the introduced combination of conditions and assumptions, conflict-induced selectivity adjustments and temporal learning predict opposite modulations of the CDPCE: While selectivity adjustments predict a larger CDPCE in the Contrasting case, temporal learning predicts a larger one in the Matching case. This means that both mechanisms predict a three-way interaction between congruency, PC, and case, across both contexts, but opposite signs for the equivalent t-test comparing the CDPCEs.

### Computational illustration

Above, we have derived the opposing predictions of the two adaptation mechanisms for idealized scenarios without variation in the relevant variables. Therefore, we neither specified that the predicted patterns should emerge at the level of mean RTs and mean error rates (ERs), nor described how they arise from single-trial processing dynamics. To illustrate the latter process in a tangible way, we implemented the introduced combination of conditions and assumptions in simulations of both conflict monitoring and temporal learning. In addition to their illustrative purpose, these simulations can be regarded as a proof of principle for our claim that an implementation of the kind of interdependence described above into common computational representations of the two mechanisms produces the predicted data patterns.

**Fig 2:**
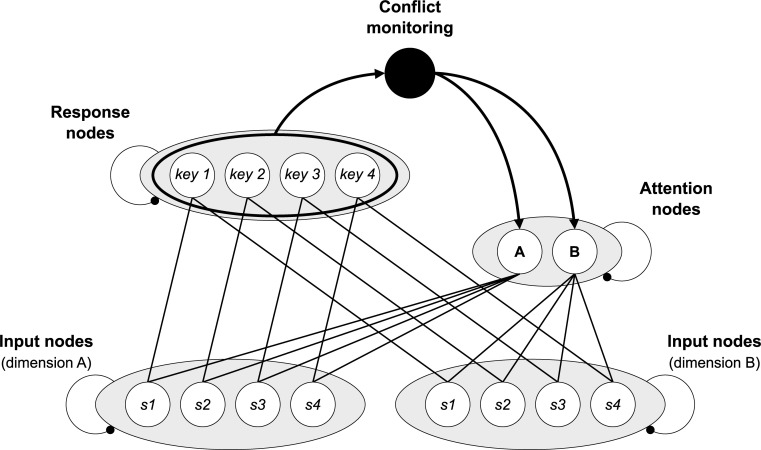
Adapted CMT model. Schematic illustration of the structure of the adapted CMT model. “s1”, “s2”, “s3”, and “s4” denote the four possible stimuli, “key 1”, “key 2”, key 3”, and “key 4” denote the four possible response. “A” and “B” denote the two stimulus dimensions.

**Fig 3:**
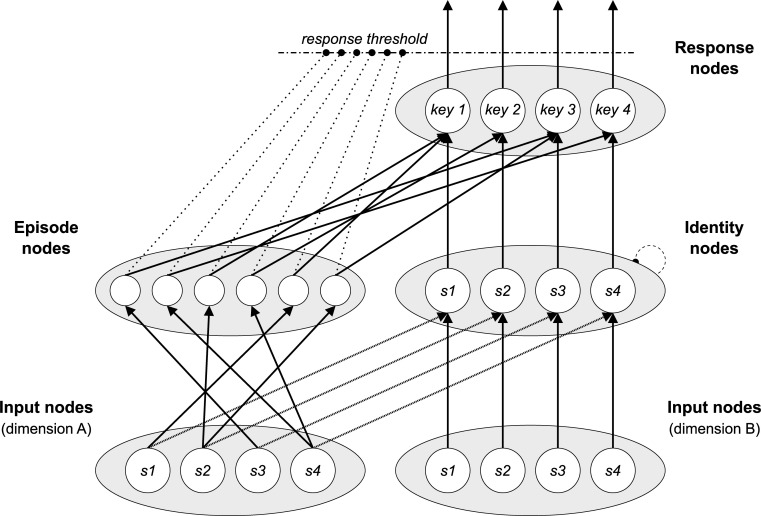
Adapted PEP model 1.2.0. Schematic illustration of the structure of the adapted PEP model 1.2.0. “s1”, “s2”, “s3”, and “s4” denote the four possible stimuli, “key 1”, “key 2”, key 3”, and “key 4” denote the four possible response.

The adapted computational models simulate information processing in experimental conflict protocols at the single-trial level, from stimulus presentation to response execution. As Figs 2 and 3 indicate, in each simulation run (i.e., each trial), “activation” is propagated iteratively through a network of interconnected nodes from input to response nodes.

### Conflict monitoring model

In 2001, Botvinick et al. [8] demonstrated that an engagement of cognitive control to the degree that response conflict is detected can explain both the CSE and the PCE. They did so by integrating the proposed conflict monitoring and control feedback mechanism into previously established models of trial-level information processing in the Stroop task [1,2,42] and the Eriksen Flanker task [3,4,43], developed within the parallel distributed processing framework.

**Fig 4:**
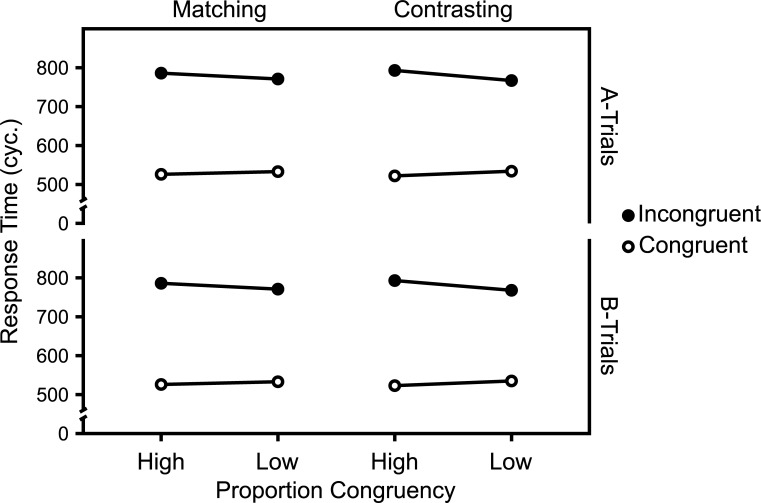
RT Predictions of the adapted CMT model. Predicted mean RTs (in cycles), in the Matching and Contrasting case, by two instantiations of the adapted CMT model simulating two task contexts (“A-Trials” and “B-Trials”) with reversed target and distractor dimensions.

We adapted a variant of the conflict monitoring model of the Stroop task [8], used by the authors for follow-up explorations. As Fig 4 shows, we simulated a task with the minimum number of stimuli required to create subsets of inducer (s1 and s2) and diagnostic (s3 and s4) stimuli. Apart from the necessary changes to the stimulus-response mapping, we only made the following structural changes to the model:

- We equated the hard-wired influence of the two stimulus dimensions (originally color and word) such that prioritization of either dimension was only achieved through task-dependent processing weights represented by the attention units (“A” and “B”, in Fig 4). We made this change due to the requirement of defining the target and distractor dimensions purely based on the task context. As we were unsure to what extent a potential global processing bias [44] would manifest when the local dimension contains the target, and in order to simulate as general a scenario as possible, we implemented no such bias.
- The basic weight of the respective target dimension was .6, and the basic weight of the respective distractor dimension was .2, in each task. The basic weights were adjusted based on the level of conflict, according to the implemented standard conflict-monitoring mechanism [8]. The maximum bias towards the target dimension, that is, the maximum weight difference that could be achieved was .8, the minimum one was .02. As in the flanker model of Botvinick et al. [8], the adjustments were compensatory such that an increase of the weight of one dimension was accompanied by an equal decrease of the weight of the other.

The complete code of the model can be found in S1 Appendix.

To simulate the switches between the two contexts, two instantiations of the model with reversed target and distractor dimensions were run in parallel, on randomly alternating simulation runs. In accordance with assumption one, each instantiation processed the trial information of one task/context almost entirely independently of the respective other instantiation. In accordance with assumption two, however, in each trial (i.e., a simulation run), the implemented adjustment of the target and distractor weights was calculated as the above introduced weighted average of the (according to the model and our hand-wired parameters) currently appropriate adjustment in the active instantiation (weighted with .75) and the currently appropriate adjustment in the respective other instantiation (weighted with .25).

For both the Matching and the Contrasting case, 10000 participants each processing 672 (336 per context/instantiation) were simulated. For each participant, the first 32 were practice trials, which featured the same context-specific PCs as the first five of the following ten experimental blocks of 64 trials each. In both the Matching and the Contrasting case, the sequence of PC conditions was counter-balanced: In the Matching case, 5000 participants began with high PC in both contexts, and 5000 participants began with low PC in both contexts. In the Contrasting case, 5000 participants began with high PC in the A-trials and low PC in the B-trials, and 5000 participants began with low PC in the A-trials and high PC in the B-trials.

**Fig 5:**
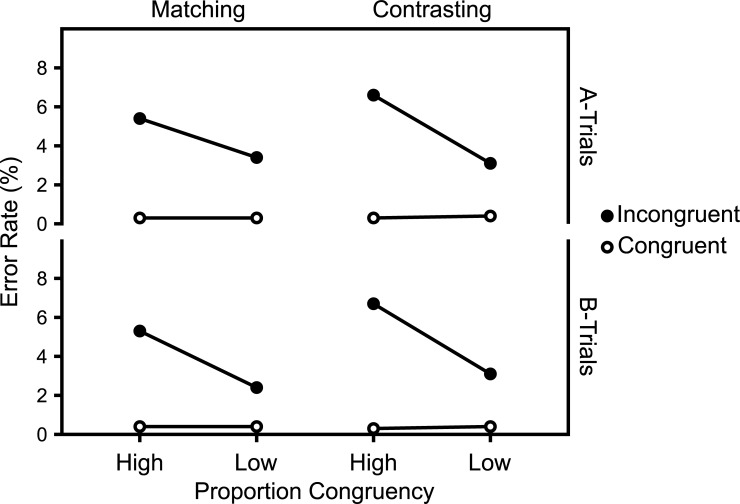
ER Predictions of the adapted CMT model. Predicted mean ERs, in the Matching and Contrasting case, by two instantiations of the adapted CMT model simulating two task contexts (“A-Trials” and “B-Trials”) with reversed target and distractor dimensions.

As Figs 4 and 5 show, the CDPCE was larger in the Contrasting case than in the Matching case, in both mean RTs and ERs. In mixed analyses of variance with Congruency, Context/Instantiation, and PC as within-subject factors and Case (Matching vs. Contrasting case) as a between-subjects factor, significant three-way interactions between Congruency, PC, and Case revealed that this difference was significant for both RTs and ERs (*p* < .001 in both cases). Note that, because the model does not behave differently for diagnostic trials than for inducer trials, all trials were used, in the ER analysis. In the RT analysis, error trials were excluded.

### Temporal learning model

In 2013, Schmidt demonstrated that temporal learning can explain the list-level PCE by integrating a temporal learning mechanism into a previously developed model of trial-level information processing in the Stroop task, the Parallel Episodic Processing Model [1,2,30]. With the latter, he had demonstrated that contingency learning via storage and retrieval of episodic memories can explain the item-specific proportion congruency effect [30].

We adapted the modified version of the PEP Model, version 1.2.0 [12]. As Fig 3 shows, we implemented the same stimulus response mapping as in the case of the CMT model. No other structural changes were made. The complete code of the model can be found in S2 Appendix.

As described above, two instantiations of the model were run in parallel, on randomly alternating simulation runs. In this case, there was no difference between the instantiations reflecting the reversal of the target and distractor dimension as the assignment of these roles is arbitrary, in the model. In each trial, the implemented (shift in) the temporal expectation was calculated as the weighted average of the currently appropriate temporal expectation in the active instantiation (weighted with .75) and the currently appropriate temporal expectation in the respective other instantiation (weighted with .25).

The same amounts of participants and trials per participants were simulated in the same way as described above, for the Matching and the Contrasting case.

**Fig 6:**
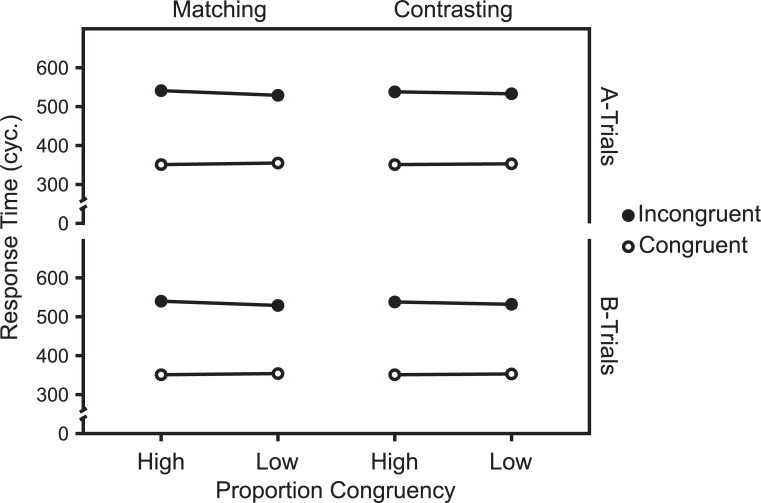
RT Predictions of the Adapted PEP Model 1.2.0. Predicted mean RTs (in cycles), in the Matching and Contrasting case, by two instantiations of an adapted version the PEP model 1.2.0 simulating two task contexts (“A-Trials” and “B-Trials”) with reversed target and distractor dimensions.

As expected, the CDPCE was larger in the Contrasting case than in the Matching case, in both mean RTs, as visualized in Fig 6, and ERs. The analyses were the same as above, except that only diagnostic trials were analyzed. The expected three-way interaction between Congruency, PC, and Case was only significant (*p* < .001) for mean RTs.

### Evaluation

In this section, we evaluate methodological and theoretical aspects of our approach for dissociating selectivity adjustments from temporal learning. First, we describe potential alternative explanations to the predicted opposite modulations of the CDPCE. Then, we discuss matters of reliability and generalizability.

### Confounds

**Fig 7:**
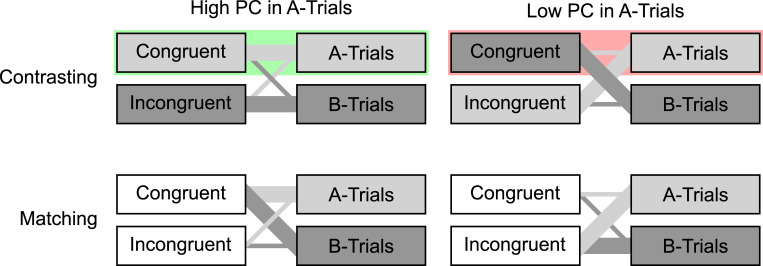
Contingencies between context and congruency. The thicknesses of the lines connecting the boxes represent the strengths of the associations between congruency levels and contexts, based on the frequencies of co-occurrence. The color of each congruency level box indicates whether the respective congruency level predicts a particular context (i.e., whether it occurs more in one context level than in the other), and, if so, which. The green background indicates that, when the PC in the A-trials is high, congruent A-trials constitute a combination of a congruency level with the context this congruency level predicts. By contrast, the red background indicates that, when the PC in the A-trials is low, congruent A-trials constitute a combination of a congruency level with another context than the one this congruency level predicts.

Hypothetically, there could be cases in which selectivity adjustments are not the only possible explanation for a larger CDPCE in the Contrasting case. Fig 7 illustrates learned associations that could provide an alternative.

In each context, a PCE indicates a processing advantage of trials with the currently more frequent congruency level. In the Contrasting case, an explanation for this advantage could be that the congruency level predicts the correct context in those trials but the wrong one in trials with the less frequent congruency level.

For example, when the PC in the A-trials is high, the more frequent congruency level of congruent A-trials (marked green) correctly predicts an A-trial. By contrast, when the PC in the A-trials is low, the less frequent congruency level of congruent A-trials (marked red) wrongly predicts a B-trial.

Thus, in each context, predictions of the context based on the congruency level could induce a PCE, in the Contrasting case. By contrast, in the Matching case, no such predictions are possible because the congruency level is never predictive of either context. This difference could explain a larger CDPCE in the Contrasting case.

However, note that this confound can only constitute a problem in cases in which it is likely that the congruency level can be identified sufficiently quickly to influence the identification of the context. When context cues are basic visual features like color [26,29] or location [24,25,36], but the identification of congruency demands more elaborate processing (for example in Stroop-like tasks), this is not likely. Furthermore, in all cases in which the context is cued in advance, this confound should be entirely irrelevant.

As we have seen, the one explanation of a larger CDPCE in the Contrasting case that does not involve selectivity adjustments is only plausible in very particular cases. By contrast, there are two generally applicable explanations of a larger CDPCE in the Matching case that do not involve temporal learning, as discussed below.

As Fig 1 shows, in the Matching case, the list-wide PC is manipulated in addition to the context-specific PCs, but not in the Contrasting case. Therefore, only in the Matching case, there could be a mechanism operating at the level of the list in addition to a mechanism operating within each context, thus increasing the CDPCE, in that case.

The other generally applicable explanation of a larger CDPCE in the Matching case that does not involve temporal learning is closely related to the predictive value of the congruency level, illustrated in Fig 7. This predictive value of the congruency level in the Contrasting case is due to the fact that each target-distractor combination that constitutes an inducer stimulus occurs much more often in a particular context. Therefore, in inducer trials, the target-distractor combination predicts the more frequently associated context/target level and response. James Schmidt alerted us to the fact that this predictive value of the target-distractor combination, in the majority of trials, may induce participants to generally process the stimulus constellations more holistically. We suspect that such a presumably more even distribution of processing resources would interfere more strongly with a highly selective setting than it would facilitate a highly unselective setting. Therefore, we suspect that if the processing style is affected in this way, the PCE in each context, and therefore the CDPCE, will be reduced, in the Contrasting case.

Given these two generally applicable alternative explanations for a larger CDPCE in the Matching case, the approach we introduce here is much better suited for providing evidence for selectivity adjustments than it is for providing evidence for temporal learning.

### Reliability and generalizability

#### Reliability

Reliability problems are:

- While the procedure for ruling out specific mechanisms requires eight, our approach requires 32 conditions (16 when collapsing across contexts). If not accompanied by a proportional increase in trials, this increase in conditions reduces the trials per condition which reduces reliability.
- The final measure is not the difference of a difference (two-way interaction of PC and congruency), but the difference of a difference of a difference (three-way interaction of Case, PC and congruency), for each context. This increases variability, which reduces reliability.

**Fig 8:**
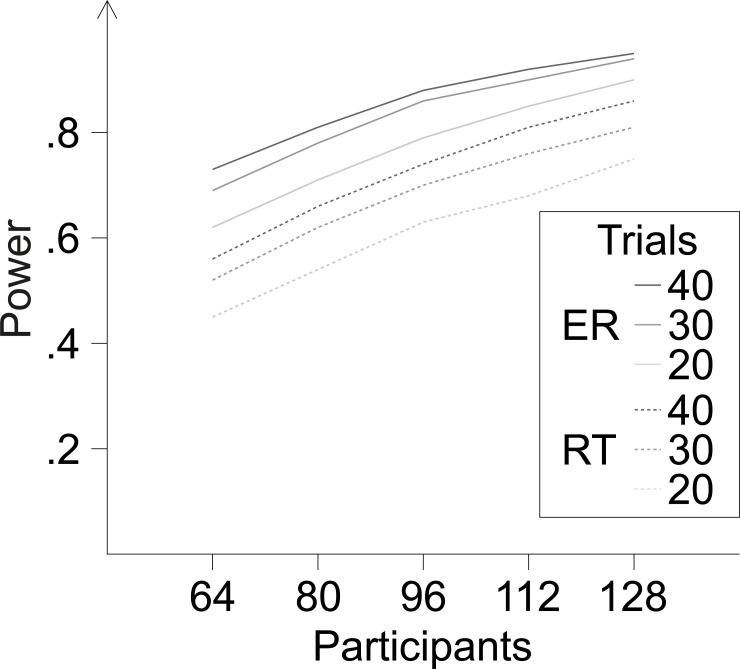
Power estimations based on bootstrapping. Estimated power for finding a CDPCE (i.e., a three-way interaction between case, congruency and PC across both context) at an alpha level of .05 for RTs (dashed lines) and ERs (solid lines) as a function of the numbers of participants (on the x-axis) and the number of diagnostic trials (legend) per condition. Each estimate is based on 10000 bootstrap iterations.

Fig 8 shows how estimated power develops as a function of the numbers of diagnostic trials per condition and of participants. For these estimations, we adapted the simulation-based method developed by Crump and Brosowsky (2019) [45] and recommended by Braem et al. [11] for power calculations for conflict protocols. We adapted this approach by extending the factorial design implemented in the function pc_modulation_power_fast() to include the factor context, by bootstrapping from distribution parameter sets based on the sample data reported below instead of using the same parameter set (per condition) for all subjects, and by estimating ER effects in addition to RT effects. The power estimations with our bootstrapping procedure are more conservative (i.e., lower) than with the procedure implemented by Crump and Brosowsky [45] and more conservative than estimations based on simple bootstrapping from the sample.

### Generalizability

With respect to generalizability, two questions can be distinguished. One question is how well the presented approach can be applied to the different types of experimental conflict protocols. Another question is to what extent the results of a particular implementation in one type of protocol allow conclusions about mechanisms that operate in other protocols or outside the laboratory.

With respect to the first question, the definition of the contexts and the process of switching between them are central. In our view, the contexts should be as similar as possible. Ideally, they should only differ in which stimulus dimension contains the target and which the distractor (and in an easily processable context cue). This should maximize the probability that the same adaptation mechanism operates in both contexts and the probability that the respective context-specific adaptations influence each other. Other factors that likely increase these probabilities, especially if one assumes that carry-over effects are the cause of the interdependence, are the unpredictability of context switches and short preparation times for the new context during switches. These specifications limit the range of protocols in which the presented approach can be readily implemented. While, for example, a reversal of the target and distractor dimensions has often even been part of the Stroop task itself [1,2], it cannot be applied in the Simon task [46]. In the latter task, a dimension reversal would also have to involve a change of the target and distractor categories.

Based on these considerations, it could be argued that any conclusions drawn from the implementation of the approach in a particular protocol only apply to those protocols in which the approach is equally well applicable. In fact, it could be argued that such conclusions only apply even more narrowly, namely to the modified version of those protocols which the implementation of the approach would create (i.e., versions involving switches between two contexts with reversed target and distractor dimensions).

The latter restriction, however, violates the principle of parsimony as it supposes that different adaptation mechanisms or different versions of the same mechanisms operate in a context when it is presented in isolation than when it is presented in combination with another very similar context. A similar argument can be made against the first restriction. To the extent that the different experimental conflict protocols can be said to measure the same adaptation mechanisms, conclusions about the operation of one of those mechanisms in one of those protocols should be generalizable to other protocols. Otherwise, one would have to assume different versions of the same mechanisms for each protocol. Especially with respect to selectivity adjustments, the different experimental conflict protocols, also known as interference protocols, are traditionally considered to measure the same mechanisms, at some level of abstraction.

## Illustrative implementation

In this section, we report two experiments that, though not designed as such, constitute an implementation of the abstract conditions. Due to their favorable results, these experiments are well-suited to illustrate the promise of our approach for dissociating selectivity adjustments from temporal learning. At the same time, they constitute a case in which it may just be conceivable that selectivity adjustments are not the only possible explanation of a larger CDPCE in the Contrasting case. That is because congruency was marked by perceptual homogeneity (see below) and may therefore have been identified sufficiently quickly to influence the identification of the context. Thus, the experiments also illustrate this potential challenge to our approach.

**Fig 9:**
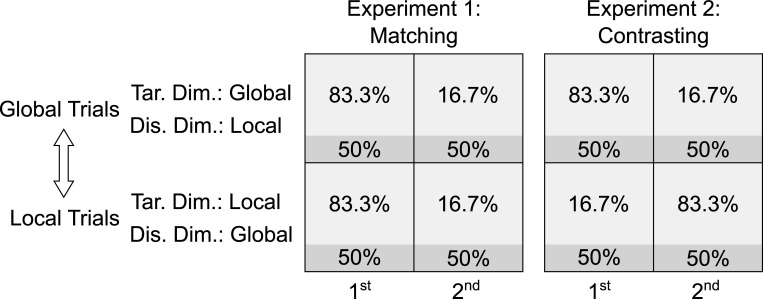
Illustrative concrete experimental conditions. The double-headed arrow indicates the switches between the “Global Trials”, each of which involved a global target, and the “Local Trials”, each of which involved a local target. “Tar.”, “Dis.” and “Dim.” stand for “Target”, “Distractor”, and “Dimension”. The percentages are target-level- and trial-type-dependent PCs: Areas in lighter gray represent inducer trials. Areas in darker gray represent diagnostic trials. The size of each area represents the proportion of trials of the respective type, in the respective quadrant (i.e., 75% are inducer trials and 25% diagnostic trials). “1^st^” and “2^nd^” stand for the first and second half of the trial sequence constituting the Matching or the Contrasting case, respectively.

- Participants switched between responding to the global and responding to the local level of hierarchical visual (Navon) stimuli.
- As Fig 9 shows, in each context, the PCs of the more frequent inducer stimuli (lighter gray) changed, while those of the less frequent diagnostic stimuli (darker gray) did not. Still, the list/context-wide PC changed from 75% in the first to 25% in the second half of the trial sequence, or vice versa, in each context. In Experiment 1 (i.e., the Matching case), the contexts had the same PC, in each half of the trial sequence. In Experiment 2 (i.e., the Contrasting case), the contexts had different PCs, in each half.

**Fig 10:**
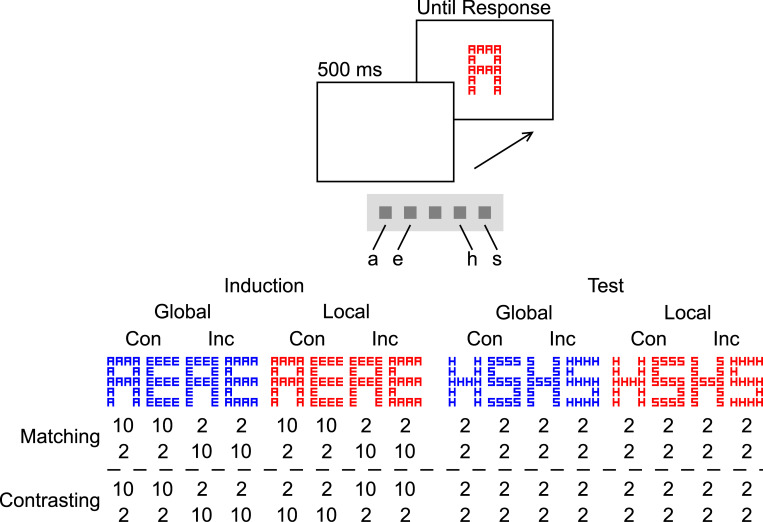
Protocol and stimulus frequencies. Each row of numbers represents the presentation frequencies in one block. Both for the Matching and the Contrasting case (i.e., Experiment 1 and 2), the upper row represents the frequencies in a block with a PC of 75% in trials involving a global target, the lower represents those in a block with a PC of 25% in trials involving a global target. “Con” and “Inc” stand for “congruent” and “incongruent”, respectively.

Fig 10 shows the protocol and block-wise presentation frequencies.

Under these conditions and the theoretical assumptions introduced in the first section, selectivity adjustments and temporal learning predict opposite modulations of the CDPCE. Exactly analogous to the abstract scenario above, selectivity adjustments predict a larger CDPCE in the Contrasting case (i.e., Experiment 2) whereas temporal learning predicts a larger one in the Matching case (i.e., Experiment 1).

### Methods

#### Participants

Thirty-two healthy students of the Medical School Hamburg (20 females and 12 males), ranging in age from 21 to 29 years, participated in Experiment 1. Twenty-nine healthy students of the Medical School Hamburg (22 females and 7 males), ranging in age from 20 to 31 years, participated in Experiment 2. Of the originally 32 participants, three had to be excluded due to a lack of valid data in one condition. These sample sizes were not based on a priori power analyses or other considerations concerning effect size. The post-hoc power estimations above show that adequate to good power can be reached by increasing the number of participants from 64 to 128 (overall) and increasing the number of trials from 672 to 992 (which increases the number of diagnostic trials per condition by a factor of 1.5 from 20 to 30). These numbers are high but still feasible.

The research and testing procedure were in accordance with all legal requirements, with the 1964 Helsinki Declaration including its later amendments, and with the ethical recommendations of the Deutsche Gesellschaft für Psychologie (DGPs; German Psychological Association). All participants gave written informed consent prior to their participation. There was no need for further approval by an ethics committee.

### Stimuli

See Fig 10. The global stimuli extended 28 mm horizontally and 44 mm vertically. The local stimuli extended 6 mm horizontally and 8 mm vertically.

### Procedure

Participants sat at a table on which the monitor presenting the stimuli (refresh rate of 60 Hz) was placed at a viewing distance of about 60 cm. Participants were instructed to rest their index and middle fingers on the four outermost buttons of a Serial Response Box (Psychology Software Tools, Inc.). As Fig 3 shows, the letter stimuli were mapped onto these buttons in alphabetical order. The participants were instructed to respond to the global letter when the hierarchical stimulus was blue and to the local letter when it was red. They were instructed to respond as quickly and accurately as possible. Each trial started with a white screen for 500 ms. Then, the hierarchical stimulus was presented at the center of the screen until the participant responded. For each trial, a hierarchical stimulus was randomly chosen (without replacement) from the respective set (see Fig 3). Each session consisted of one practice block before ten experimental blocks. The practice block consisted of 32, each experimental block of 64 trials. The target-level-specific PCs in the practice block were the same as in the first five experimental blocks. After these, the PC in trials involving a global target changed, either from 25% to 75% or vice versa (order balanced across participants). In Experiment 1, the PC in trials involving a local target was always the same as the PC in trials involving a global target. In Experiment 2, it always had the respective other value. Between blocks, participants were allowed to rest for some time. A complete session lasted about 50 minutes.

### Data analysis

We excluded data from trials associated with response times (RTs) below 200 ms or above 2500 ms. We also excluded data from the first trial of each block, the practice blocks, error trials, and trials immediately following errors. On average, we excluded 15% of data per subject for Experiment 1, and 20% of data per subject for Experiment 2.

The following analyses were computed for both mean RTs and error rates (ERs):

- First, we conducted an omnibus-analysis involving all factors of the design, namely a 2 (Experiment: Matching vs. Contrasting) x 2 (Stimulus Type: Inducer vs. Diagnostic) x 2 (Target Level: Global vs. Local) x 2 (PC: high [i.e., 75%] vs. low [i.e., 25%]) x 2 (Congruency: Incongruent vs. Congruent) mixed analysis of variance (ANOVA).
- As we were mainly interested in the diagnostic trials, we excluded the factor Stimulus Type and conducted a separate 2 (Experiment: Matching vs. Contrasting) x 2 (Target Level: Global vs. Local) x 2 (PC: high [i.e., 75%] vs. low [i.e., 25%]) x 2 (Congruency: Incongruent vs. Congruent) mixed ANOVA of data from diagnostic and in inducer trials, respectively.
- Finally, even though the CDPCE is defined across contexts (see above), we investigated whether the expected three-way interaction would manifest in each context, in the diagnostic trials, by conducting a separate 2 (Experiment: Matching vs. Contrasting) x 2 (PC: high [i.e., 75%] vs. low [i.e., 25%]) x 2 (Congruency: Incongruent vs. Congruent) mixed ANOVA of data from global and local diagnostic trials, respectively.

The complete results of these analyses can be found in Appendix B. Here, we only report the results that pertain to the issue at hand.

### Results

**Fig 11:**
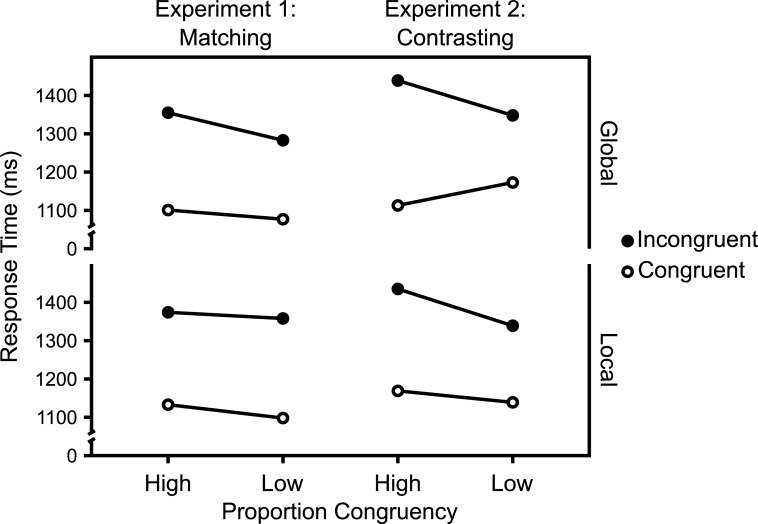
RTs in diagnostic trials. Mean RTs in diagnostic trials as a function of Experiment, Target Level, target-level-specific PC, and Congruency. N_Matching_ = 32, N_Contrasting_ = 29.

**Fig 12:**
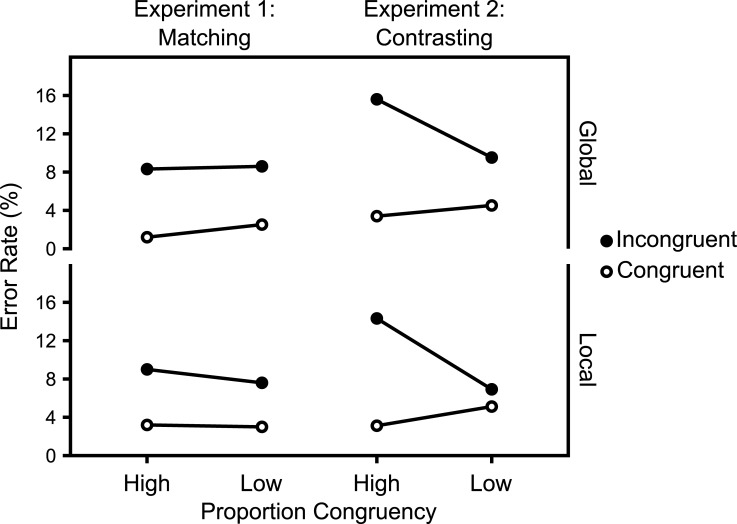
ERs in diagnostic trials. Mean ERs in diagnostic trials as a function of Experiment, Target Level, target-level-specific PC, and Congruency. N_Matching_ = 32, N_Contrasting_ = 29.

**Fig 13:**
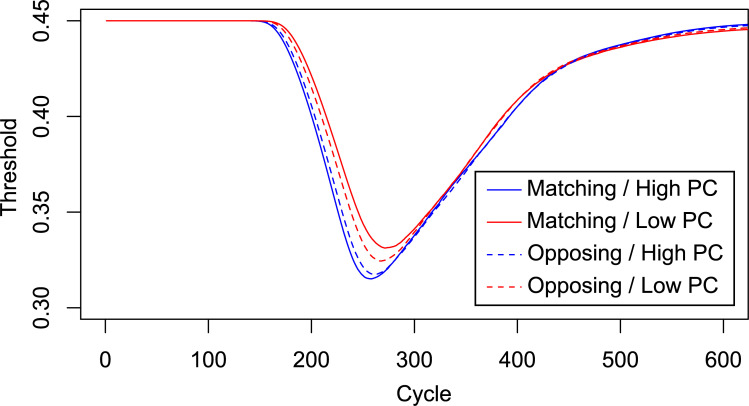
Average response thresholds. Average response threshold values of the adapted temporal learning model (adapted version of PEP model 1.2.0) as a function of simulation cycle, in a single trial. Each threshold value represents an average across 100 simulated subjects, both contexts/model instantiations, and all diagnostic trials over the course of an entire simulated experiment.

Figs 11 and 12 show the mean RTs and ERs in the diagnostic trials and Fig 13 show the Average response thresholds.

#### Overall

The 2 (Experiment: Matching vs. Contrasting) x 2 (Target Level: Global vs. Local) x 2 (PC: high [i.e., 75%] vs. low [i.e., 25%]) x 2 (Congruency: Incongruent vs. Congruent) mixed ANOVA of the mean RTs in diagnostic trials revealed a significant interaction between Experiment, PC, and Congruency, *F*(1,59) = 6.22, *p* = .015, *η*^*2*^_*g*_ = .002.

The corresponding analysis of the ERs in diagnostic trials also revealed a significant interaction between Experiment, PC, and Congruency, *F*(1,59) = 8.53, *p* = .005, *η*^*2*^_*g*_ = .012.

### Global target level

The 2 (Experiment: Matching vs. Contrasting) x 2 (PC: high [i.e., 75%] vs. low [i.e., 25%]) x 2 (Congruency: Incongruent vs. Congruent) mixed ANOVA of the mean RTs in *global diagnostic trials* revealed a marginally significant interaction between Experiment, PC, and Congruency, *F*(1,59) = 3.64, *p* = .061, *η*^*2*^_*g*_ = .002.

The corresponding analysis of the ERs in global diagnostic trials revealed no significant interaction between Experiment, PC, and Congruency, *F*(1,59) = 2.78, *p* = .101, *η*^*2*^_*g*_ = .009.

### Local target level

The 2 (Experiment: Matching vs. Contrasting) x 2 (PC: high [i.e., 75%] vs. low [i.e., 25%]) x 2 (Congruency: Incongruent vs. Congruent) mixed ANOVA of the mean RTs in *local diagnostic trials* revealed no significant interaction between Experiment, PC and Congruency, *F*(1,59) = 2.73, *p* = .104, *η*^*2*^_*g*_ = .002.

The corresponding analysis of the ERs in local diagnostic trials revealed a significant interaction between Experiment, PC, and Congruency, *F*(1,59) = 7.48, *p* = .008, *η*^*2*^_*g*_ = .016.

### Discussion

Though not planned as such, Experiments 1 and 2 can be regarded as an implementation of the abstract experimental conditions introduced in the first section. Thus, given the assumptions introduced in that section, selectivity adjustments and temporal learning predict opposite modulations of the CDPCE, in these experiments. While the former predict a larger CDPCE in the Contrasting case (i.e., Experiment 2), the latter predicts a larger one in the Matching case (i.e., Experiment 1).

The results clearly favor selectivity adjustments. As Figs 10 and 11 show, the CDPCE was larger in the Contrasting case, for both mean RTs and ERs. While the three-way interaction between Experiment, PC, and Congruency was not significant for most single combinations of target level and dependent variable, it was significant for both mean RTs and ERs when aggregated across target levels (i.e., the CDPCE).

This pattern permits the exclusion of temporal learning as the sole cause of the CDPCEs. Furthermore, in the absence of alternative explanations for a larger CDPCE in the Contrasting case, one would have to conclude that selectivity adjustments have taken place. This means that it would be possible to unequivocally attribute stimulus-unspecific adaptations to PC to selectivity adjustments–at least partially. To our knowledge, these experiments would constitute the first case in which this was possible. Note that this interpretation presupposes that there is no alternative explanation (to selectivity adjustments) for a larger CDPCE in the Contrasting case.

It could be argued, however, that the reported experiments are a case in which there is an alternative explanation. That is because congruency was marked by perceptual homogeneity (i.e., in congruent trials, the local letters were identical to the global letter). Therefore, it could be argued, it is conceivable that the congruency level could be identified sufficiently quickly to influence the identification of the target level–even though the context was cued by the basic feature color. If so, predictions of the target level based on the congruency level may have contributed to the CDPCE in the Contrasting case, but not in the Matching case (see the subsection Confound in the first section).

However, as the context was cued by the basic feature color, we do not consider this likely. In any case, the fact that the results of these particular experiments could potentially be explained in a different way does not diminish the value of our approach in general. That is because, as we have pointed out, there are many cases in which the confound cannot provide a viable alternative explanation: The most straightforward ways to create such scenarios may be advance cueing of the context, and using protocols in which congruent and incongruent stimulus configurations do not differ perceptually.

## Conclusion

The list-level PCE and the CSPC effect can only be regarded as indicators of stimulus-unspecific conflict-induced selectivity adjustments [8] if all other mechanisms can be ruled out as their sole cause. Currently, temporal learning as their sole cause can only be ruled out statistically, but not experimentally. That is because, in all established protocols designed to quantify the traditional list-level PCE or CSPC effect, temporal learning makes exactly the same prediction as selectivity adjustments do.

Here, we have presented a step towards solving this problem and experimentally dissociating selectivity adjustments from temporal learning. To this end, we have introduced an as yet unexplored hybrid form of the list-level PCE and the CSPC effect, the context-dependent PCE (CDPCE). This effect also quantifies stimulus-unspecific adaptations to PC, and it is especially well suited to reveal reciprocal influences between contexts. Our novel approach is designed to exploit such influences. It consists in a combination of abstract experimental conditions and theoretical assumptions given which selectivity adjustments and temporal learning predict opposite modulations of the CDPCE, as we illustrated with two computational models. With experimental protocols that implement these abstract conditions, it is therefore possible, to rule out temporal learning as the sole cause of stimulus-unspecific adaptations to PC, if the CDPCE is modulated in the way that selectivity adjustments predict. Moreover, unless the conditions are implemented in a very particular way, it is also possible, to unequivocally attribute the observed CDPCEs to selectivity adjustments, at least partially.

As an illustrative implementation, we have reported two experiments whose results permit the exclusion of temporal learning as the sole cause though arguably not the selectivity adjustment interpretation. However, as we have suggested what types of protocol would also permit the selectivity adjustment interpretation, the potential limitation of the presented implementation does not diminish the value of our approach in general: To our knowledge, it currently represents the only case in which selectivity adjustments and temporal learning predict opposite outcomes. Therefore, in our view, it is worthy of further exploration.

## Supporting information

S1 Table (DOCX)

S1 Appendix Conflict monitoring model. (ZIP)

S2 Appendix Temporal learning model. (ZIP)
