# Supplementary material for: Dissociating selectivity adjustments from temporal learning–introducing the context-dependent proportion congruency effect
Source: PLoS One. 2022 Dec 13;17(12):e0276611. doi: 10.1371/journal.pone.0276611 (PMC9747054; doi:10.1371/journal.pone.0276611)
Supplement: S1 Table — (DOCX) [file pone.0276611.s001.docx]

**Appendix B**

Tables 1 and 2 show the results of the ANOVAs of the mean RTs and ERs in all trials.

**Table 1**

*ANOVA of the Mean RTs in all Trials*

| Predictor | *df_Num_* | *df_Den_* | *F* | *p* | *η^2^_g_* |
| --- | --- | --- | --- | --- | --- |
| Experiment (E) | 1 | 59 | 0.64 | .428 | .007 |
| Congruency (C) | 1 | 59 | 281.80 | <.001 | .212 |
| PC | 1 | 59 | 4.29 | .043 | .005 |
| Target Level (L) | 1 | 59 | 2.35 | .131 | .002 |
| Trial Type (T) | 1 | 59 | 279.46 | <.001 | .151 |
| E x C | 1 | 59 | 0.13 | .719 | <.001 |
| E x PC | 1 | 59 | 1.11 | .297 | .001 |
| E x L | 1 | 59 | 3.44 | .068 | .002 |
| E x T | 1 | 59 | 0.51 | .478 | <.001 |
| C x PC | 1 | 59 | 83.35 | <.001 | .016 |
| C x L | 1 | 59 | 2.15 | .148 | <.001 |
| PC x L | 1 | 59 | 0.01 | .929 | <.001 |
| C x T | 1 | 59 | 0.39 | .535 | <.001 |
| PC x T | 1 | 59 | 0.95 | .333 | <.001 |
| L x T | 1 | 59 | 0.03 | .865 | <.001 |
| E x C x PC | 1 | 59 | 0.01 | .940 | <.001 |
| E x C x L | 1 | 59 | 0.71 | .403 | <.001 |
| E x PC x L | 1 | 59 | 0.60 | .440 | .001 |
| E x C x T | 1 | 59 | 0.89 | .351 | <.001 |
| E x PC x T | 1 | 59 | 6.39 | .014 | .001 |
| E x L x T | 1 | 59 | 0.75 | .391 | <.001 |
| C x PC x L | 1 | 59 | 5.83 | .019 | .001 |
| C x PC x T | 1 | 59 | 22.67 | <.001 | .004 |
| C x L x T | 1 | 59 | 5.42 | .023 | .001 |
| PC x L x T | 1 | 59 | 0.29 | .594 | <.001 |
| E x C x PC x L | 1 | 59 | 1.58 | .213 | <.001 |
| E x C x PC x T | 1 | 59 | 12.74 | .001 | .002 |
| E x C x L x T | 1 | 59 | 0.56 | .456 | <.001 |
| E x PC x L x T | 1 | 59 | 0.33 | .565 | <.001 |
| C x PC x L x T | 1 | 59 | 0.34 | .565 | <.001 |
| E x C x PC x L x T | 1 | 59 | 1.34 | .251 | <.001 |

**Table 2**

*ANOVA of the ERs in all Trials*

| Predictor | *df_Num_* | *df_Den_* | *F* | *p* | *η^2^_g_* |
| --- | --- | --- | --- | --- | --- |
| Experiment (E) | 1 | 59 | 6.05 | .017 | .026 |
| Congruency (C) | 1 | 59 | 119.09 | <.001 | .208 |
| PC | 1 | 59 | 30.32 | <.001 | .030 |
| Target Level (L) | 1 | 59 | 3.71 | .059 | .004 |
| Trial Type (T) | 1 | 59 | 2.88 | .095 | .002 |
| E x C | 1 | 59 | 4.25 | .044 | .009 |
| E x PC | 1 | 59 | 11.03 | .002 | .011 |
| E x L | 1 | 59 | 0.75 | .391 | .001 |
| E x T | 1 | 59 | 0.03 | .864 | <.001 |
| C x PC | 1 | 59 | 48.23 | <.001 | .046 |
| C x L | 1 | 59 | 4.60 | .036 | .005 |
| PC x L | 1 | 59 | 0.05 | .818 | <.001 |
| C x T | 1 | 59 | 15.16 | <.001 | .007 |
| PC x T | 1 | 59 | 21.22 | <.001 | .009 |
| L x T | 1 | 59 | 5.36 | .024 | .003 |
| E x C x PC | 1 | 59 | 15.43 | <.001 | .015 |
| E x C x L | 1 | 59 | 0.10 | .749 | <.001 |
| E x PC x L | 1 | 59 | 0.15 | .704 | <.001 |
| E x C x T | 1 | 59 | 5.34 | .024 | .002 |
| E x PC x T | 1 | 59 | 1.05 | .309 | <.001 |
| E x L x T | 1 | 59 | 0.54 | .464 | <.001 |
| C x PC x L | 1 | 59 | 1.68 | .200 | .002 |
| C x PC x T | 1 | 59 | 14.61 | <.001 | .006 |
| C x L x T | 1 | 59 | 0.36 | .549 | <.001 |
| PC x L x T | 1 | 59 | 5.70 | .020 | .002 |
| E x C x PC x L | 1 | 59 | 0.03 | .856 | <.001 |
| E x C x PC x T | 1 | 59 | 0.19 | .665 | <.001 |
| E x C x L x T | 1 | 59 | 0.03 | .856 | <.001 |
| E x PC x L x T | 1 | 59 | <0.01 | .963 | <.001 |
| C x PC x L x T | 1 | 59 | 8.05 | .006 | .003 |
| E x C x PC x L x T | 1 | 59 | 1.00 | .320 | <.001 |

Tables 3 and 4 show the results of the ANOVAs of the mean RTs and ERs in all diagnostic trials.

**Table 3**

*ANOVA of the Mean RTs in all Diagnostic Trials*

| Predictor | *df_Num_* | *df_Den_* | *F* | *p* | *η^2^_g_* |
| --- | --- | --- | --- | --- | --- |
| Experiment (E) | 1 | 59 | 0.77 | .384 | .009 |
| Congruency (C) | 1 | 59 | 209.71 | <.001 | .186 |
| PC | 1 | 59 | 4.21 | .045 | .006 |
| Target Level (L) | 1 | 59 | 1.77 | .189 | .002 |
| E x C | 1 | 59 | <0.01 | .969 | <.001 |
| E x PC | 1 | 59 | 0.01 | .934 | <.001 |
| E x L | 1 | 59 | 1.25 | .269 | .001 |
| C x PC | 1 | 59 | 9.97 | .003 | .003 |
| C x L | 1 | 59 | 0.02 | .902 | <.001 |
| PC x L | 1 | 59 | 0.08 | .772 | <.001 |
| E x C x PC | 1 | 59 | 6.22 | .015 | .002 |
| E x C x L | 1 | 59 | 1.11 | .296 | <.001 |
| E x PC x L | 1 | 59 | 0.92 | .342 | .001 |
| C x PC x L | 1 | 59 | 4.15 | .046 | .001 |
| E x C x PC x L | 1 | 59 | 0.06 | .802 | <.001 |

**Table 4**

*ANOVA of the ERs in all Diagnostic Trials*

| Predictor | *df_Num_* | *df_Den_* | *F* | *p* | *η^2^_g_* |
| --- | --- | --- | --- | --- | --- |
| Experiment (E) | 1 | 59 | 4.43 | .040 | .023 |
| Congruency (C) | 1 | 59 | 82.21 | <.001 | .149 |
| PC | 1 | 59 | 4.03 | .049 | .006 |
| Target Level (L) | 1 | 59 | 0.04 | .847 | <.001 |
| E x C | 1 | 59 | 1.04 | .311 | .002 |
| E x PC | 1 | 59 | 4.47 | .039 | .007 |
| E x L | 1 | 59 | 1.15 | .289 | .002 |
| C x PC | 1 | 59 | 13.01 | .001 | .019 |
| C x L | 1 | 59 | 2.09 | .153 | .003 |
| PC x L | 1 | 59 | 0.73 | .396 | .001 |
| E x C x PC | 1 | 59 | 8.53 | .005 | .012 |
| E x C x L | 1 | 59 | 0.14 | .714 | <.001 |
| E x PC x L | 1 | 59 | 0.13 | .725 | <.001 |
| C x PC x L | 1 | 59 | 0.17 | .683 | <.001 |
| E x C x PC x L | 1 | 59 | 0.14 | .713 | <.001 |

Tables 5 and 6 show the results of the ANOVAs of the mean RTs and ERs in all inducer trials.

**Table 5**

*ANOVA of the Mean RTs in all Inducer Trials*

| Predictor | *df_Num_* | *df_Den_* | *F* | *p* | *η^2^_g_* |
| --- | --- | --- | --- | --- | --- |
| Experiment (E) | 1 | 59 | 0.43 | .513 | .005 |
| Congruency (C) | 1 | 59 | 271.62 | <.001 | .244 |
| PC | 1 | 59 | 3.33 | .073 | .004 |
| Target Level (L) | 1 | 59 | 1.80 | .184 | .002 |
| E x C | 1 | 59 | 0.55 | .461 | .001 |
| E x PC | 1 | 59 | 4.62 | .036 | .005 |
| E x L | 1 | 59 | 4.82 | .032 | .004 |
| C x PC | 1 | 59 | 101.41 | <.001 | .041 |
| C x L | 1 | 59 | 7.36 | .009 | .002 |
| PC x L | 1 | 59 | 0.01 | .908 | <.001 |
| E x C x PC | 1 | 59 | 6.18 | .016 | .003 |
| E x C x L | 1 | 59 | 0.08 | .781 | <.001 |
| E x PC x L | 1 | 59 | 0.24 | .624 | <.001 |
| C x PC x L | 1 | 59 | 3.13 | .082 | .001 |
| E x C x PC x L | 1 | 59 | 3.79 | .056 | .001 |

**Table 6**

*ANOVA of the ERs in all Inducer Trials*

| Predictor | *df_Num_* | *df_Den_* | *F* | *p* | *η^2^_g_* |
| --- | --- | --- | --- | --- | --- |
| Experiment (E) | 1 | 59 | 6.56 | .013 | .029 |
| Congruency (C) | 1 | 59 | 116.74 | <.001 | .273 |
| PC | 1 | 59 | 57.13 | <.001 | .072 |
| Target Level (L) | 1 | 59 | 9.66 | .003 | .016 |
| E x C | 1 | 59 | 6.97 | .011 | .022 |
| E x PC | 1 | 59 | 12.59 | .001 | .017 |
| E x L | 1 | 59 | 0.08 | .776 | <.001 |
| C x PC | 1 | 59 | 70.34 | <.001 | .085 |
| C x L | 1 | 59 | 4.48 | .038 | .008 |
| PC x L | 1 | 59 | 1.74 | .193 | .003 |
| E x C x PC | 1 | 59 | 14.13 | <.001 | .018 |
| E x C x L | 1 | 59 | 0.03 | .869 | <.001 |
| E x PC x L | 1 | 59 | 0.11 | .744 | <.001 |
| C x PC x L | 1 | 59 | 7.32 | .009 | .009 |
| E x C x PC x L | 1 | 59 | 0.51 | .477 | .001 |

Tables 7 and 8 show the results of the ANOVAs of the mean RTs and ERs in the *global diagnostic trials*.

**Table 7**

*ANOVA of the Mean RTs in the Global Diagnostic Trials*

| Predictor | *df_Num_* | *df_Den_* | *F* | *p* | *η^2^_g_* |
| --- | --- | --- | --- | --- | --- |
| Experiment (E) | 1 | 59 | 1.25 | .268 | .015 |
| Congruency (C) | 1 | 59 | 128.64 | <.001 | .174 |
| PC | 1 | 59 | 1.51 | .224 | .004 |
| E x C | 1 | 59 | 0.23 | .630 | <.001 |
| E x PC | 1 | 59 | 0.35 | .554 | .001 |
| C x PC | 1 | 59 | 12.86 | .001 | .009 |
| E x C x PC | 1 | 59 | 3.64 | .061 | .002 |

**Table 8**

*ANOVA of the ERs in the Global Diagnostic Trials*

| Predictor | *df_Num_* | *df_Den_* | *F* | *p* | *η^2^_g_* |
| --- | --- | --- | --- | --- | --- |
| Experiment (E) | 1 | 59 | 5.88 | .018 | .037 |
| Congruency (C) | 1 | 59 | 53.66 | <.001 | .175 |
| PC | 1 | 59 | 0.52 | .472 | .002 |
| E x C | 1 | 59 | 0.90 | .347 | .004 |
| E x PC | 1 | 59 | 2.72 | .104 | .009 |
| C x PC | 1 | 59 | 4.36 | .041 | .014 |
| E x C x PC | 1 | 59 | 2.78 | .101 | .009 |

Tables 9 and 10 show the results of the ANOVAs of the mean RTs and ERs in the *local diagnostic trials*.

**Table 9**

*ANOVA of the Mean RTs in the Local Diagnostic Trials*

| Predictor | *df_Num_* | *df_Den_* | *F* | *p* | *η^2^_g_* |
| --- | --- | --- | --- | --- | --- |
| Experiment (E) | 1 | 59 | 0.31 | .581 | .004 |
| Congruency (C) | 1 | 59 | 215.56 | <.001 | .199 |
| PC | 1 | 59 | 2.95 | .091 | .008 |
| E x C | 1 | 59 | 0.29 | .590 | <.001 |
| E x PC | 1 | 59 | 0.56 | .456 | .002 |
| C x PC | 1 | 59 | 0.73 | .395 | .001 |
| E x C x PC | 1 | 59 | 2.73 | .104 | .002 |

**Table 10**

*ANOVA of the ERs in the Local Diagnostic Trials*

| Predictor | *df_Num_* |  | *df_Den_* | *F* | *p* | *η^2^_g_* |
| --- | --- | --- | --- | --- | --- | --- |
| Experiment (E) | 1 |  | 59 | 1.49 | .228 | .012 |
| Congruency (C) | 1 |  | 59 | 42.61 | <.001 | .123 |
| PC | 1 |  | 59 | 4.26 | .043 | .014 |
| E x C | 1 |  | 59 | 0.35 | .556 | .001 |
| E x PC | 1 |  | 59 | 1.50 | .225 | .005 |
| C x PC | 1 |  | 59 | 11.17 | .001 | .024 |
| E x C x PC | 1 |  | 59 | 7.49 | .008 | .016 |
