# Supplementary material for: Dissociating selectivity adjustments from temporal learning–introducing the context-dependent proportion congruency effect
Source: PLoS One. 2022 Dec 13;17(12):e0276611. doi: 10.1371/journal.pone.0276611 (PMC9747054; doi:10.1371/journal.pone.0276611)
Supplement: S2 Appendix — (ZIP) [file pone.0276611.s003.zip › S2 Appendix/Description.docx]

**Model Changes**

As indicated in the main article, the only structural change we made to the PEP model 1.2.0 is that we reduced both the number of targets and the number of responses from six to four, respectively. Here, we describe the only other change, which was necessary to implement the assumed reciprocal influence with respect to the implemented (shifts in the) temporal expectations.

As described in the introduction of the main article, according to the temporal learning account [12], temporal expectations are reflected in the development of the response threshold which is transiently reduced in a particular time window during each trial. To describe how the response threshold is determined, in the original PEP model 1.2.0, in each simulation cycle, Schmidt [12] states

“…the response threshold in the model drops as the cycle time (i.e., simulated millisecond of the trial) approaches the stored response times of previously encountered trials. In addition, the influence of a given episode is decreased the longer ago it occurred. More concretely, the response threshold on a given processing cycle was determined with the formula,

$threshold=baseline-\left( \sum_{i=1}^{n} \left( proximity_{i}\times strength_{i} \right)-.01 \right)$ (1)

The *threshold* is restrained between the *baseline* threshold of .45 and a minimum drop to .30. Note that the threshold only drops after the summed (*proximity* x *strength*) scores exceed .01. The proximity of an episode *i* is determined with the formula,

$proximity_{i}=1-\left( \frac{\left( cycle-rt_{i} \right)^{2}}{10000} \right)$ (2)

The given *proximity* value of each episode is restricted between 0 and 1. This formula works such that when the current cycle time (*cycle*) is very close to the stored response time of the episode (*rt*), there is a large effect on the response threshold. The further apart they are, the more rapidly the influence of the node drops off (and no longer has an influence with a difference of 100 or more cycles). The strength of episode *i* is determined by the formula,

$strength_{i}=\frac{\left( 40-lag_{i} \right)^{2}}{50000}$ (3)

In this formula, *lag* represents how many trials previously a given node *i* occurred. These formulas are only applied to the most recent 40 trials, so *strength* can vary from .03042 (i.e., the most recent episode) down to 0 (i.e., episodes from 40 trials back or more). Note that the influence of a node is decreased logarithmically the further ago it occurred, such that the most recent episode has an especially large impact, and this influence decreases at a decelerating rate for increasingly longer lags.” [12].

As described in the main article, we ran two instantiations of the adapted version of the PEP model 1.2.0. in parallel, on randomly alternating simulation runs (i.e., trials). In each cycle of each simulation run, the influence of memory-based expectations on the response threshold, which determines to what extent the response threshold is reduced in that cycle (see above) – and thereby the development of the threshold over the course of the trial, namely the value of

$$bias_{active instantiation}=\left( \sum_{i=1}^{n} \left( proximity_{i}\times strength_{i} \right)-.01 \right)$$

in the active model instantiation is weighted with .75, and added to the value of the same variable in the currently inactive instantiation

$$bias_{inactive instantiation}=\left( \sum_{i=1}^{n} \left( proximity_{i}\times strength_{i} \right)-.01 \right)$$

weighted with .25. Thus, the current threshold was determined by:

$$threshold=baseline-(bias_{active instantiation}\times.75+ bias_{inactive instantiation}\times.25)$$

The consequence of this is that the dip in the response threshold is mostly influenced by the expectation(s) generated by the currently active instantiation but also slightly by the expectation(s) generated by the currently inactive instantiation.

The result of this reciprocal influence is visualized in Figure 2 (below), which shows average response threshold developments (over the course a trial), for 100 simulated subjects per condition, averaged across both contexts. As described formally on pages 12 to 13 of the main article, in the matching condition (solid lines), the difference in the temporal expectations (i.e., in the shapes of the threshold developments) between the PC conditions is larger (in each context) than in the opposing condition (dashed lines). Therefore, the simulated PCE (in each context) is larger in the matching condition than in the opposing condition.


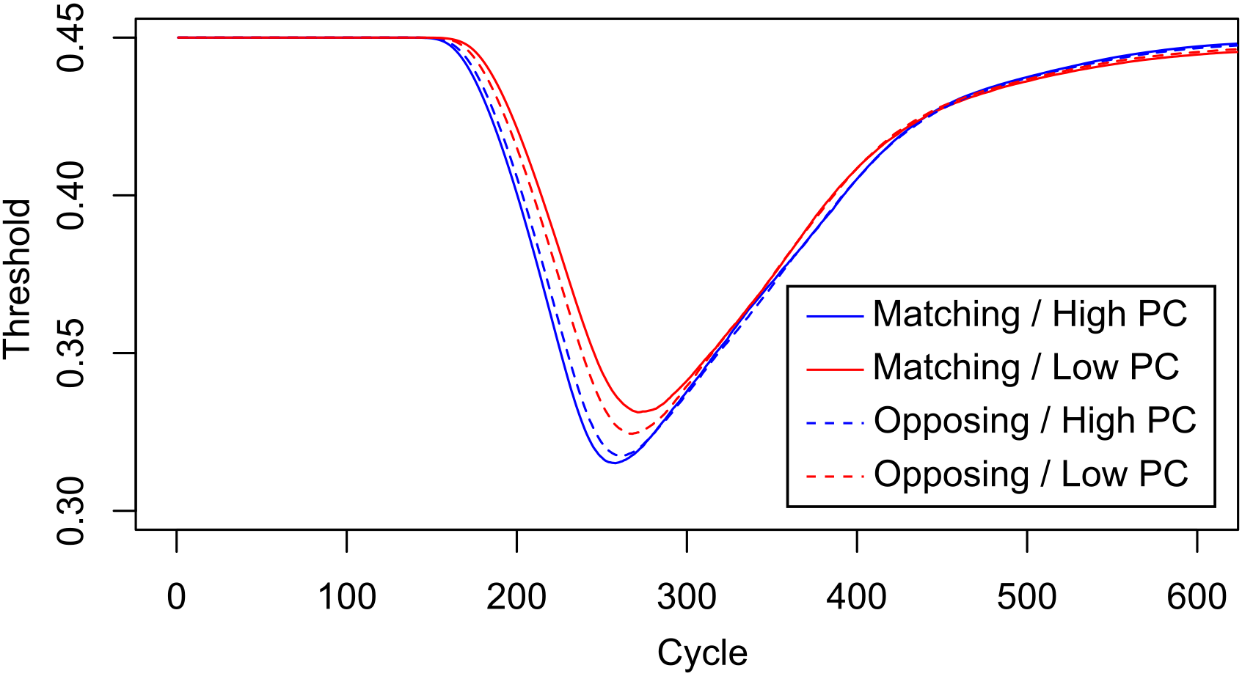


**Figure 13. Average Response Thresholds.** Average response threshold values of the adapted temporal learning model (adapted version of PEP model 1.2.0) as a function of simulation cycle, in a single trial. Each threshold value represents an average across 100 simulated subjects, both contexts/model instantiations, and all diagnostic trials over the course of an entire simulated experiment.
